# In Vitro Activity of Mupirocin Against Methicillin-Resistant *Staphylococcus aureus* (MRSA) in Two Periods of Time (2016 and 2021–2022) in a Large University Hospital in Germany

**DOI:** 10.3390/microorganisms14030650

**Published:** 2026-03-13

**Authors:** Arved Carl Christian Westphal, Mandy Vogel, Catalina Suzana Stingu

**Affiliations:** 1 Institute for Medical Microbiology and Virology, University of Leipzig Medical Center, 04103 Leipzig, Germany; arved.westphal@medizin.uni-leipzig.de; 2 German Centre for Child and Adolescent Health (DZKI), Partner Site Leipzig/Dresden, 04103 Leipzig, Germany; mandy.vogel@medizin.uni-leipzig.de

**Keywords:** mupirocin, MRSA, prevalence of mupirocin resistance, mupirocin activity

## Abstract

Colonization with methicillin-resistant *Staphylococcus aureus* (MRSA) increases the risk of adverse health outcomes, and it is estimated that 10–30% of carriers subsequently develop infection. Mupirocin is currently widely used as a topical decolonization measure against nasal methicillin-resistant *S. aureus* (MRSA) carriers. The present study was carried out in order to determine the prevalence rate of high-level (HLMuR) and low-level (LLMuR) mupirocin-resistant MRSA strains among patients treated at our hospital over two periods of time (2016 and 2021–2022) and to observe possible changes in MRSA susceptibilities against mupirocin after a six-year period of use. This is a retrospective study carried out on MRSA isolated from various clinical specimens from inpatients. A total of 474 MRSA isolates found in nasal, nasopharyngeal, throat, wound, urine and prostheses swabs were examined. All *S. aureus* isolates were identified using MALDI-TOF mass spectrometry scores ≥ 2 and confirmed as MRSA using the cefoxitin (30 µg) disc diffusion method. Mupirocin-resistant MRSA was detected by the Epsilometer test (E-test). All the resistant strains were tested for Panton-Valentine-Leucocidin (PVL) toxin by PCR. Out of 481 strains in our database, 474 pure non-duplicate MRSA isolates were included in our study. Mupirocin resistance was found in 15 (3.2%) MRSA strains by E-test, whereby three (0.6%) isolates were HLMuR and 12 (2.5%) isolates were LLMuR. The prevalence of mupirocin-resistant MRSA was similar in 2016 (10.6%) and 2021 (12.9%) and decreased to 6.5% in 2022. Even though the decrease in the proportion of resistant isolates from 2021 to 2022 did not reach statistical significance (*p* = 0.103), the mean resistance level among resistant isolates decreased significantly over the study period (*p* ≤ 0.001), dropping from 94.3 µg/ml in 2016 to 46.4 µg/ml in 2021 with a further decrease to 0.7 µg/ml in 2022. Although the mupirocin resistance decreased in 2022 compared with 2016, continuous monitoring of mupirocin resistance development in MRSA and surveillance of excessive use is of utmost importance in order to rule out possible failure of mupirocin decolonization treatment with subsequent increased mortality due to infections, prolonged hospital stays and more difficult treatment requirements at an early stage.

## 1. Introduction

*Staphylococcus aureus* is a leading cause of healthcare-associated infections worldwide and is associated with increased morbidity, mortality and healthcare costs [1]. Colonization with methicillin-resistant *S. aureus* (MRSA) increases the risk of adverse health outcomes, and it is estimated that 10–30% of carriers subsequently develop infection [1,2]. The nasal as well as extra-nasal sites such as the throat, axillary or inguinal colonization with *S. aureus* generally precedes invasive infection [1,3]. A systematic review concluded that MRSA colonization conferred a 4-fold increased risk of infection as compared with methicillin-sensitive *S. aureus* (MSSA) colonization [3,4,5,6,7,8]. Eradication of MRSA carriage from the nose and other body sites forms an integral part of strategies to prevent and control MRSA in many countries [9]. Soon after the first MRSA was identified in 1960, ß-lactam antibiotics were shown to be less effective due to the mecA gene encoding a modified penicillin-binding protein, called PBP2a, which leads to a lower affinity for ß-lactam antibiotics and consequently methicillin resistance in staphylococci [10]. As a result, mupirocin, which is an important component in MRSA prevention and decolonization, has been increasingly used specifically for the eradication of nasal MRSA in patients and healthcare personnel and sometimes even for *S. aureus* skin and soft tissue infections (e.g., Impetigo) [1,10,11,12,13]. Mupirocin is a fermentation product of *Pseudomonas fluorescens*. It has in vitro activity against staphylococci, some streptococci, *Enterococcus faecium* and *Haemophilus influenzae*. The effect of mupirocin is based on the inhibition of protein biosynthesis of the pathogen by blocking leucine-specific-tRNA aminoacyl synthetase, which prevents the amino acid Isoleucine from binding to the tRNA [10,12,14]. However, reports of increasing mupirocin resistance after extended and widespread use are of serious concern, which is significantly associated with persistent mupirocin-resistant MRSA carriage [1,10]. According to EUCAST, mupirocin resistance appears in two phenotypes: low-level mupirocin resistance (LLMuR) (MIC 8–256 µg/ml) and high-level mupirocin resistance (HLMuR) (MIC > 256 µg/ml) [12,14,15,16,17]. Low resistance is caused by point mutations in the ileS gene, while high resistance is caused by plasmids carrying the mupA gene [1,18,19]. It is known that topical application of mupirocin is not effective against MRSA strains with high-level resistance and sometimes even low-level resistance [10].

The aim of this study was to determine the prevalence of mupirocin resistance over two time periods (2016 and 2021–2022) and to observe possible changes in MRSA susceptibilities against mupirocin after a six-year period of use.

Therefore, we tested our hypothesis that the prevalence of mupirocin resistance has increased during the current mupirocin application from 2016 to 2021 and 2022 in a large university hospital in Germany.

## 2. Materials and Methods

### 2.1. Selection of Strains

All the MRSA clinical strains found in the nasal, nasopharyngeal, throat and wound swabs in 2016 and 2021–2022 at the Institute for Medical Microbiology and Virology, Department of Medical Microbiology, were included in this study. Duplicate strains, as well as strains from blood culture, were excluded to focus primarily on indication-related topical areas of mupirocin application.

The strains were stored at −80 °C using the cryotubes (Mast CRYOBANK^®^, Bootle, UK). All strains were then grown on blood agar plates (bioMérieux^®^, Marcy-l’Étoile, France) for 24 h at 36–37 °C. The identification of all strains was performed using the MALDI Biotyper system (Bruker^®^ Daltonics, Bremen, Germany). All the strains were identified with a score ≥ 2.00 [20].

**Figure 1:**
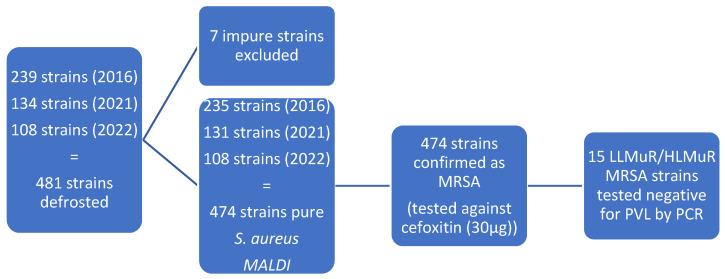
Flowchart outlining the selection steps for the LLMuR and HLMuR MRSA strains.

The disk diffusion method with cefoxitin discs was used to confirm the methicillin resistance of the strains. All *Staphylococcus aureus* strains with inhibition zones smaller than 22 mm were confirmed as MRSA ([21], Figure 1).

### 2.2. Mupirocin Susceptibility by MIC Strip Tests

Müller Hinton agar plates were incubated with a bacterial suspension of turbidity equivalent to 0.5 McFarland (McF) standard. Minimum Inhibitory Concentration (MIC) Strip Tests (bioMérieux^®^ E-test) with mupirocin antibiotic ranges from <0.064 to >1024 µg/ml were then applied with sterile forceps, and the plates were incubated for 18–24 h at 36 °C under aerobic conditions. The MIC-reading was performed using the reading guide provided by the manufacturer [22,23,24]. The interpretation of the results was done using the document by the European Committee on Antimicrobial Susceptibility Testing (EUCAST): Mupirocin Rationale for the EUCAST clinical breakpoints [14]. The MRSA strains were categorized according to their MIC values into sensitive (MIC < 4 µg/ml), low-level mupirocin-resistant (LLMuR, MIC 8–256 µg/ml) and high-level mupirocin-resistant (HLMuR, MIC > 256 µg/ml) [14].

Low- and high-level resistant MRSA strains with a MIC between 8 and 256 µg/ml or >256 µg/ml were also compared by year 2016, 2021 or 2022.

### 2.3. Correlation PVL-Status by PVL PCR

All the LLMuR and HLMuR MRSA strains were tested for the production of Panton-Valentine-Leucocidin (PVL) by the lukS-PV gen, using PVL Multiplex PCR.

We used MRSA cultures of the LLMuR and HLMuR strains for the PVL LightMix^®^ Multiplex PCR testing as the sample material in combination with LightMix^®^ Modular CA-MRSA ReagentMix (TibMolBiol, Berlin, Germany). The sample preparation was carried out with 500 µl MagNA Pure Bacteria Lysis Buffer (Roche^®^, Basel, Switzerland) and two to three resistant MRSA cultures which were mixed and tested on a 200 µl processing plate. The reaction batch consisted of 15 µl MasterMix containing 10 µl PCR-H_2_O, 4 µl Roche Master solution, 0.5 µl CA-MRSA ReagentMix, 0.5 µl PhHV ReagentMix and 5 µl MRSA strain sample DNA gained through volume transfer with MagNA Pure Bacteria Lysis Buffer (Roche^®^). Detection according to PVL was carried out with the LightCycler^®^ 480 II Instrument, the LightCycler^®^ 480 Software (Version LCS480 1.5.1.62) and Hexaplex CC (21 July 2014) as colour compensation. All samples except the positive control were PVL-negative when we performed the Multiplex PCR test.

### 2.4. Statistical Analysis

The prevalence (P) of mupirocin-resistant MRSA strains within the corresponding years was determined by the quotient of the number of resistant strains and the number of all strains within the corresponding years [Table 1].

We compared the distributions of resistance levels between 2016, 2021, and 2022 using generalized models for location, shape, and scale, assuming a zero-adjusted gamma distribution (ZAGA). A ZAGA distribution was chosen because the MIC data exhibit two distinct features that simpler approaches cannot accommodate simultaneously: (1) a large proportion of isolates (~75–88%) are susceptible (MIC < 0.064 µg/ml, recoded as zero), creating a point mass at zero, and (2) among isolates with MIC ≥ 0.064 µg/ml, MIC values are continuous and right-skewed. The ZAGA model decomposes the analysis into a binary (logistic) component comparing the proportion of isolates with ≥0.064 µg/ml vs. susceptible isolates across years, and a continuous (gamma) component comparing the mean resistance level among isolates above the lower detection limit. A simple chi-square test or Fisher’s exact test would only address the proportion of resistance, discarding information about the magnitude of resistance, whereas a standard continuous model (e.g., ANOVA on MIC values) would be inappropriate given the extreme zero-inflation and non-normality of the data.

The ZAGA model allows us to compare the percentages of MRSA-positive (resistant, with varying levels), zero (susceptible/no clinically relevant resistance) and the level of resistance for the non-negative samples simultaneously. Since E-test MIC values are continuous and never yield a true zero, all isolates with MIC values measured as <0.064 µg/ml were recoded as zero (i.e., susceptible/no clinically relevant resistance). All MIC values ≥ 0.064 µg/ml were retained as continuous positive values. This dichotomization into a zero component (susceptible) and a positive component (resistant, with varying levels) forms the basis of the zero-adjusted gamma distribution model. The predictions for resistance and level of resistance were reported as percentages and means. The effects were reported as odds ratios (OR, comparison of percentages) and ratios (exp(ß), comparison levels of resistance).

## 3. Results

**Figure 2:**
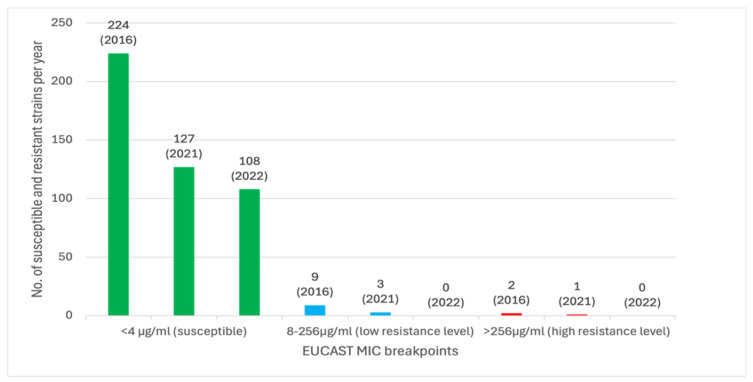
Number of susceptible and resistant strains per year according to EUCAST.

A total of 474 MRSA isolates were included in the study, with 235 strains from 2016, 131 strains from 2021, and 108 strains from 2022. The strains were collected from the nasopharyngeal cavity (233 strains, 49.16%), nose cavity (17 strains, 3.59%), wounds (219 strains, 46.20%), urine samples (3 strains, 0.63%), and prostheses (2 strains, 0.42%). The results of the mupirocin testing are summarized in the [Table 1] and [Figure 2]. Mupirocin resistance was found in 15 (3.2%) MRSA strains by E-test, whereby three (0.6%) isolates were HLMuR and 12 (2.5%) isolates were LLMuR [Figure 2].

**Figure 3:**
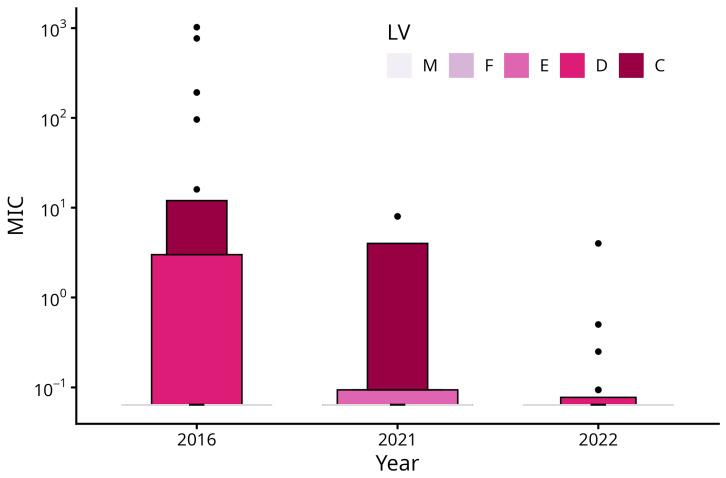
Letter-value plot showing the distribution of minimum inhibitory concentrations (MIC) by year. M (median) and F (fourths) collapsed due to the highly right-skewed distribution, consisting mainly of lower MIC values. The median and the surrounding M and F boxes collapse to the detection limit and are not visually distinguishable from the baseline. These levels are therefore represented by a thin line at the bottom of the plot rather than a visible box.

The prevalence of mupirocin-resistant MRSA was similar in 2016 (10.6%) and 2021 (12.9%) and decreased to 6.5% in 2022. However, despite the considerable effect size (OR = 0.47), the decrease from 2021 to 2022 did not reach statistical significance (*p* = 0.103). Moreover, we found a consistent trend in the gamma component of the distribution (i.e., the location trend in the non-zero values), with a decrease from 94.3 µg/ml in 2016 to 46.4 µg/ml in 2021 (exp(ß) = 0.49, *p* = 0.284) and a further decrease to 0.7 µg/ml in 2022 (exp(ß) = 0.02, *p* ≤ 0.001). Likewise, the prevalence of high-level resistant strains was similar in 2016 and 2021 (0.85% vs. 0.76%). In 2022, there were neither high- nor low-level resistant strains. Figure 3 (letter-value plot) is showing the distribution of minimum inhibitory concentrations (MIC) by year. Due to the high share of low MIC values, only the upper portions of letter values E (87.5th–93.8th percentiles), D (93.8th–96.9th percentiles), and C (96.9th–98.4th percentiles) are visible as colored boxes, with more extreme values displayed as individual points. The y-axis is presented on a logarithmic scale. Letter values M (median) and F (fourths) collapsed due to the highly right-skewed distribution, consisting mainly of lower MIC values [Figure 3].

According to our results, an increase in mupirocin resistance in tested MRSA strains from 2016 to 2021 and 2022 could therefore be ruled out. The significance level of our study was set to ⍺ = 0.05.

## 4. Discussion

The importance of nasal carriage of staphylococcal strains, especially MRSA, has been long recognized. Often, strains causing infections are the ones which colonize the patient’s nose [5,25,26]. Use of mupirocin has also been associated not only with eradication of nasal carriage but also eradication in other sites of the body [11,13]. It also appears to be one of the most effective topical regimes so far [27,28]. It has also demonstrated efficacy in decolonization strategies within clinical settings. However, resistance to mupirocin is emerging, and this could consequently lead to underlining the importance of surveillance and eventually to the necessity of developing alternative strategies for eradication of *S. aureus* carriage [29,30,31,32].

The emergence of antimicrobial resistance poses a significant challenge to public health, necessitating accurate and efficient antimicrobial susceptibility testing (AST) methods [33,34,35]. The Clinical and Laboratory Standards Institute (CLSI) and the European Committee on Antimicrobial Susceptibility Testing (EUCAST) have established guidelines to standardize these testing methods. The interpretation of the results in this study was done using the EUCAST Guidelines. Isolates with MICs > 256 µg/ml were considered HLMuR, those with MICs 8–256 µg/ml were considered LLMuR and those with <4 µg/ml were considered mupirocin-susceptible [14].

The estimated prevalence of mupirocin-resistant MRSA in Europe was 14.1% with a high-level mupirocin-resistant prevalence in MRSA of 8.0% [10]. It is noticeable that the prevalence of mupirocin resistance in MRSA (MuRMRSA) worldwide has risen from 10.5% before 2006 to 23.8% in 2015, and the prevalence of high level mupirocin resistance in MRSA (HLMuRMRSA) increased from 8.2% before 2006 to 14.3% in 2015 [10]. In comparison to Asia (12.1%) and the USA (5.9%), Europe’s (8.0%) HLMuRMRSA resistance situation is relatively moderate [10]. It should be noted, thereafter, that mupirocin resistance in clinical isolates of *S. aureus* varies depending on the geographic region, the patient population, the misuse of antibiotics, and the ease of access to over-the-counter antibiotics [10]. This shows that one of the most effective antibiotics to decontaminate the nasal MRSA is becoming a case of concern due to increased resistance in some regions of the world.

With an estimated prevalence of MuRMRSA in the USA of 15.1% in total and a HLMuR-MRSA prevalence in MRSA of 5.9%, the USA places at the top with regard to high-level mupirocin-resistant MRSA in international comparison [10]. The decolonization treatment in the USA consists of nasal application of mupirocin twice daily for 5–10 days [36].

In contrast, the estimated MuRMRSA prevalence in Asia is the lowest at 13.4%, but the HLMuRMRSA prevalance of 12.1% is the highest in international comparison [10]. In particular, MRSA prevalence in India may range between 32 and 80% among the *S. aureus* strains and has increased by 37% in the observation period from 2015 to 2020 [37,38]. This is caused by overuse of antimicrobial drugs; increase in infections due to lack of sanitation, hygiene and general awareness of the arbitrary use of antibiotics; as well as deficiency of stringent rules and regulations for use of antibiotics [38]. For MRSA eradication in nasal carriers’, mupirocin topical application five times daily is the method of choice in India [39].

The national surveillance data from China Antimicrobial Resistance Surveillance System (CARSS) showed a median prevalence rate of MRSA during 2014–2019 between 23.1 and 46.9% [40,41]. Mupirocin has gained attention in the treatment of severe skin and soft tissue infection (SSTIs) caused by MRSA in China during the last decades. Therefore it was widely used since 1993, and in 2005, it was changed from prescription drug to an over-the-counter drug for the treatment of skin infections (e.g., impetigo, furunculosis, folliculitis) as well as eczema with infection and superficial trauma combined with infection [42]. It must be taken into account that long-term and large-area topical application of mupirocin may promote drug resistance, which is unlikely with short-term use. The positive effects of reducing MRSA colonization and cross-transmission should therefore not be compromised due to emergence of resistance such as point mutations in the ileS gene, which encodes isoleucyl-tRNA synthetase. Amino acid substitution, e.g., V588F, V631F, G593V, R816C, H67Q and F563L in IleRSs, lead to low-level mupirocin resistance, whereas high-level mupirocin resistance is plasmid-encoded [42]. Until now mupirocin has shown total low resistance in clinical applications in China, with values from 0.9 to 10% from 2004 to 2020 for MRSA with a relatively low change in mupirocin resistance rate over the years [42].

The aim of this study was to determine the prevalence of mupirocin resistance over two periods of time (2016 and 2021–2022) and to observe possible changes in MRSA resistance after a six-year period of mupirocin use in a large university hospital in Germany.

The hypothesis of our study was that the prevalence of mupirocin resistance has increased during the current mupirocin application from 2016 to 2021 and 2022.

Because the number of MRSA in 2022 was significantly lower as compared to 2016, we decided to add another year, namely 2021, to our study, in order to compare an approximately similar number of MRSA strains.

Fortunately, the prevalence of high-level resistance (HLMuRMRSA) (MIC of >256 µg/ml) decreased from 0.85% (2/235 strains) in 2016 to 0.76% (1/131 strains) in 2021 and 0% in 2022, whereas the prevalence of low-level resistance (LLMuRMRSA) (MIC of 8–256 µg/ml) was 3.83% (9/235 strains) in 2016, 2.29% (3/131 strains) in 2021 and 0% in 2022. The LLMuR MRSA or HLMuR MRSA strains were found in the nasopharyngeal cavity (4 strains), wound swaps (9 strains) and the nose (2 strains), which is congruent with the topical mupirocin application. In 2022 all MRSA strains have been mupirocin-susceptible.

Although the prevalence in our statistical analysis does not show statistical significance due to the low overall count of LLMuR and HLMuR MRSA cases from 2016 to 2022 in our study, the proven trend of declining mupirocin resistance can also be assumed to be consistent with other current comparable research findings. Due to the declining number of MRSA cases in Germany since 2017, the necessary use of mupirocin for decolonization regimens could be reduced [43,44,45,46]. The decreased prevalence of MRSA in our hospital reflects the dynamic of the MRSA prevalence in Germany. Consequently, our hypothesis was not confirmed. Taking into consideration the potentially life-threatening development of mupirocin resistance in MRSA in other countries, these results are very encouraging.

According to the prevalence surveillance study from Germany (Hospital Surveillance System—KISS), the prevalence of MRSA in Germany reached its maximum in 2012 with 1.04 cases per 100 patients, remaining at this level until 2016 before decreasing significantly in 2017 to 0.72 (−32%) and to 0.54 (−50%) by 2021 during an observation period from 2006 to 2021 [43].

Over the observed period, Germany has consistently witnessed a substantial decline in the proportion of MRSA among all *S. aureus* isolates, from 9.1% in 2017 to 4.9% in 2021 [46]. This value is notably lower than the corresponding value for the European population-weighted mean with a percentage of 15.8%, which has also undergone a substantial decrease over the past five years. Notwithstanding these encouraging developments, it is noteworthy that 30% of European countries continue to report MRSA rates of ≥25% [44]. These countries include for example Italy, Portugal, Croatia, Greece, Serbia, Turkey, Cyprus and Romania [46].

We focused on our study primarily on MRSA strains from the nose, the nasopharyngeal cavity and wounds, those being the sites where the mupirocin ointment for decolonization is applied.

We have chosen 2016 as baseline in our study because starting in 2016 the hygiene protocols in our hospital changed, with mupirocin being recommended for local nasal and mouth MRSA decolonization. As mupirocin testing is not a part of routine microbiological diagnostic, there was no local monitoring of the susceptibility of MRSA against mupirocin for this observation period until 2022.

The standard operating procedure regarding MRSA screening and decolonization at our hospital since 2016 includes MRSA screening at patient admission for those who meet the indication, such as previous MRSA infection, transfer from other care facilities or home care to our hospital, admission or planned transfer to an intensive care unit, dialysis patients, transplant recipients or patients with chronic wounds and indwelling catheters as well as persons with occupational contact to cattle. Required sample swabs are obtained from the nose, throat or chronical wounds and culturally examined. MRSA-positive patients have to perform hand disinfection after body wash, use of toilet, teeth brushing and blowing one’s nose. MRSA decolonization also contains surface disinfection of patients surroundings, removal or change in catheters, usage of disposable washcloths, and daily change of bed linen, patients’ clothes, toothbrushes and care products, as well as body wash with Octenisan^®^ washing lotion. Additionally, nasal ointment containing mupirocin (e.g.,Turixin^®^) should be applied three times daily for five days in combination with a mouth rinse solution containing chlorhexidine (e.g., Dynexan^®^) for mouth and throat decolonization.

Following decolonization treatment and/or antibiotic therapy at our hospital, control swabs must be requested as a culture and performed at the earliest 48 h after the completion of decolonization measures. During the hospital stay control swabs from nose, throat or wounds will be taken once a week.

Mupirocin has been used as the standard in other European countries, such as the Netherlands (within the programme “search and destroy” since 1988), Norway, Sweden and Finland. These countries achieved some of the lowest MRSA prevalences in the world with 0.9% in Norway, 1.5% in the Netherlands, 2.0% in Sweden and 2.6% in Finland in 2021 [25,46,47,48,49,50,51,52].

The decline in MRSA prevalence in Germany since 2017 is closely linked to the consistent implementation of the hygiene measures recommended by the Commission for Hospital Hygiene and Infection Prevention (KRINKO), including early risk-based screening, mandatory MRSA reporting, isolation protocols, and structured decolonization procedures in German hospitals including using mupirocin nasal ointment, chlorhexidine mouthwash, antiseptic body wash, and daily change of bed linen, towels, as well as daily items and personal care products [43,44,45,53].

Due to the significance of increased pathogenicity, we tested our LLMuR and HLMuR MRSA strains for PVL, as this pore-forming toxin destroys immune cells, especially leukocytes such as monocytes and neutrophil granulocytes, causing further immune deficiency and higher infectivity. PVL-positive MRSA strains are responsible for recurrent SSTIs and invasive disease (e.g., necrotizing pneumonia) [54,55,56].

All mupirocin-resistant MRSA strains from 2016, 2021 and 2022 showed negative PVL results. A potential explanation could be the low frequency of PVL-encoding genes in *S. aureus* between approximately 1–2% in nasal swab examinations. Nonetheless, the PVL gene frequency in *S. aureus* of wound swab examinations is more than 20% [53]. It is worth mentioning that systematic screening and reporting of PVL in *S. aureus* are neither mandatory nor uniformly established in Germany, with one exception: Saxony where the detection of PVL-positive MRSA has to be notified to the local health authority, when evidence indicates acute infection [55,57].

Limitations of our study are the relative low number of MRSA strain, and the fact that we only investigated MRSA strains from one university hospital in Germany, which therefore primarily reflects the local epidemiological situation.

## 5. Conclusions

Although the actual level of mupirocin resistance of the MRSA strains is low in our hospital, continuous monitoring of mupirocin resistance development in MRSA and surveillance of excessive use is of utmost importance in order to rule out possible failure of mupirocin decolonization treatment with subsequent increased mortality due to infections, prolonged hospital stays and more difficult treatment requirements at an early stage. The epidemiological patterns observed in 2022 remain only applicable to the current clinical setting if further investigation confirms the trend in declining mupirocin resistance and surveillance of long-term decolonization treatment is constantly implemented in the future. Moreover, routine in vitro mupirocin susceptibility testing seems reasonable for validation of MRSA decolonization success and early detection of treatment failure, in connection with minimizing the risk of MRSA reinfection by adhering to strict isolation and hygiene measures in hospitals.

## Figures and Tables

**Table 1:** In vitro activity of mupirocin against MRSA clinical strains in two periods of time (2016 and 2021–2022) according to EUCAST breakpoints.

MRSA + Year	No. of Strains Tested + Percentage %	MIC (Range <0.064–1024 µg/ml)										Susceptible MIC ≤4 µg/ml	Low-Level Resistant (LLR) MIC 8–256 µg/ml	High-Level Resistant (HLR) MIC >256 µg/ml
			≤4	6	8	12	16	96	192	256	>256			
2016	235	No	224	2	1	2	1	1	2	0	2	224	9	2
	% 100	%	95.32	0.85	0.43	0.85	0.43	0.43	0.85	0	0.85	95.32	3.83	0.85
2021	131	No	127	0	2	0	0	0	0	1	1	127	3	1
	% 100	%	96.95	0	1.53	0	0	0	0	0.76	0.76	96.95	2.29	0.76
2022	108	No	108	0	0	0	0	0	0	0	0	108	0	0
	% 100	%	100	0	0	0	0	0	0	0	0	100	0	0

## Data Availability

The original contributions presented in this study are included in the article. Further inquiries can be directed to the corresponding author.

## Abbreviations

The following abbreviations are used in this manuscript:

MRSA	Methicillin-resistant Staphylococcus aureus
HLMuR	High-level mupirocin-resistant
LLMuR	Low-level mupirocin-resistant
MALDI	Matrix-Assisted Laser Desorption/Ionization
TOF	Time-of-Flight
MuR	Mupirocin-resistant
PVL	Panton-Valentine-Leukocidin
MSSA	Methicillin sensitive Staphylococcus aureus
CLSI	Clinical and Laboratory Standards Institute
EUCAST	European Committee on Antimicrobial Susceptibility Testing
CARSS	China Antimicrobial Resistance Surveillance System
KRINKO	Commission for Hospital Hygiene and Infection Prevention
KISS	Hospital Infection Surveillance System
MIC	Minimum inhibitory concentration
AST	Antimicrobial Susceptibility Testing
SSTI	Severe Skin and Soft Tissue Infection
McF	McFarland
PCR	Polymerase chain reaction
OR	Odds ratio
P	Prevalence
ZAGA	Zero-adjusted gamma
